# Impaired Synaptic Plasticity and Motor Learning in Mice with a Point Mutation Implicated in Human Speech Deficits

**DOI:** 10.1016/j.cub.2008.01.060

**Published:** 2008-03-11

**Authors:** Matthias Groszer, David A. Keays, Robert M.J. Deacon, Joseph P. de Bono, Shweta Prasad-Mulcare, Simone Gaub, Muriel G. Baum, Catherine A. French, Jérôme Nicod, Julie A. Coventry, Wolfgang Enard, Martin Fray, Steve D.M. Brown, Patrick M. Nolan, Svante Pääbo, Keith M. Channon, Rui M. Costa, Jens Eilers, Günter Ehret, J. Nicholas P. Rawlins, Simon E. Fisher

**Affiliations:** 1 Wellcome Trust Centre for Human Genetics, University of Oxford, Roosevelt Drive, Oxford OX3 7BN, United Kingdom; 2 Department of Experimental Psychology, University of Oxford, South Parks Road, Oxford OX1 3UD, United Kingdom; 3 Department of Cardiovascular Medicine, University of Oxford, John Radcliffe Hospital, Headington, Oxford OX3 9DU, United Kingdom; 4 Laboratory for Integrative Neuroscience, National Institute on Alcohol Abuse and Alcoholism, National Institutes of Health, 5625 Fishers Lane, Room TS-20D, MSC 9411, Bethesda, Maryland 20852-9411; 5 Institute of Neurobiology, University of Ulm, 89069 Ulm, Germany; 6 Carl-Ludwig-Institute for Physiology, University of Leipzig, 04103 Leipzig, Germany; 7 Max-Planck Institute for Evolutionary Anthropology, Deutscher Platz 6, 04103 Leipzig, Germany; 8 Medical Research Council Mammalian Genetics Unit, Harwell, Didcot, Oxfordshire OX11 0RD, United Kingdom

**Keywords:** SYSNEURO

## Abstract

The most well-described example of an inherited speech and language disorder is that observed in the multigenerational KE family, caused by a heterozygous missense mutation in the *FOXP2* gene [1]. Affected individuals are characterized by deficits in the learning and production of complex orofacial motor sequences underlying fluent speech and display impaired linguistic processing for both spoken and written language [2]. The FOXP2 transcription factor is highly similar in many vertebrate species, with conserved expression in neural circuits related to sensorimotor integration and motor learning [3, 4]. In this study, we generated mice carrying an identical point mutation to that of the KE family, yielding the equivalent arginine-to-histidine substitution in the Foxp2 DNA-binding domain. Homozygous R552H mice show severe reductions in cerebellar growth and postnatal weight gain but are able to produce complex innate ultrasonic vocalizations. Heterozygous R552H mice are overtly normal in brain structure and development. Crucially, although their baseline motor abilities appear to be identical to wild-type littermates, R552H heterozygotes display significant deficits in species-typical motor-skill learning, accompanied by abnormal synaptic plasticity in striatal and cerebellar neural circuits.

## Results and Discussion

### An Allelic Series of Mice Carrying *Foxp2* Point Mutations

**Figure 1:**
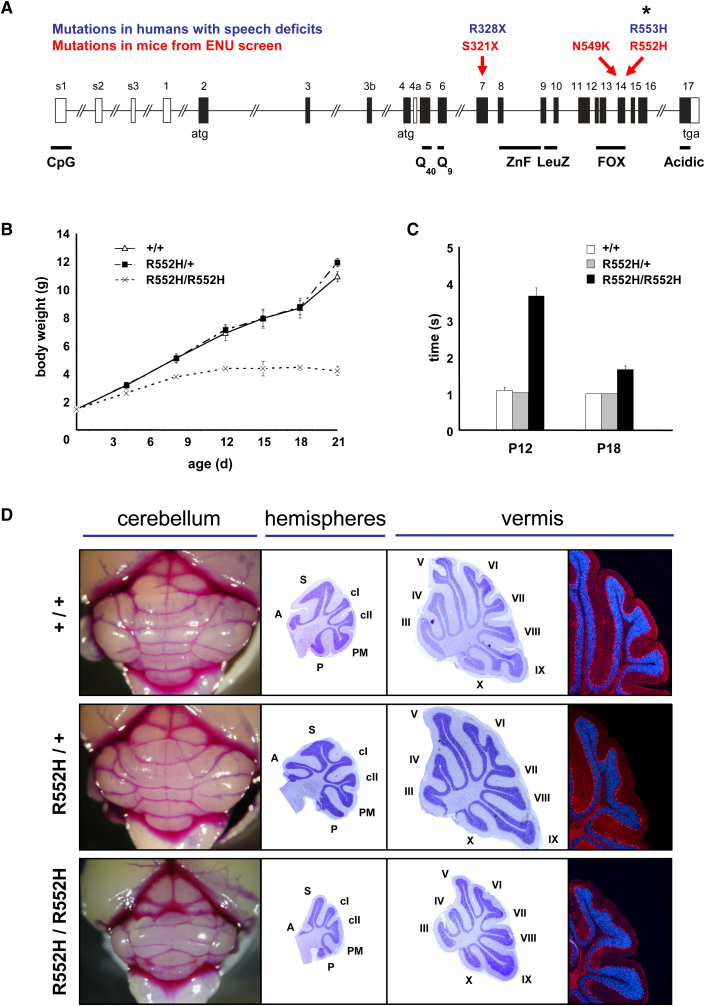
*Foxp2-R552H* Mutant Mice (A) Allelic series for mouse *Foxp2*. Mutations from gene-driven ENU mutagenesis screens are shown above a schematic of the murine *Foxp2* locus, spanning more than 500 kb of genomic DNA. Superimposed are *FOXP2* point mutations found in humans with speech and language disorders. Asterisk indicates the KE family mutation. Murine Foxp2 is one amino acid shorter than human FOXP2 because of a shorter polyglutamine tract (Q9 instead of Q10); hence, R552H in mice corresponds to R553H in humans. (B) Time course of postnatal bodyweight development. Homozygous R552H mice (n = 5) show a strongly reduced weight gain, despite feeding normally and receiving similar maternal care to littermates. Development of heterozygotes (n = 13) is indistinguishable from that of wild-type littermates (n = 8) (mean ± standard error of the mean [SEM]). (C) Postnatal righting-reflex development. Homozygous R552H mice (n = 8) display a significantly delayed righting reflex. Heterozygotes (n = 15) are indistinguishable from wild-type littermates (n = 4) (mean ± SEM). (D) Cerebellar morphology at postnatal day 21 in wild-type (top row), heterozygous (middle), and homozygous R552H mice (bottom). Homozygotes display reduced cerebellar size (left-hand column) and foliation deficits in hemispheres and vermis (middle columns, cresyl violet staining). Nevertheless, Purkinje cells are aligned in a monolayer (right-hand column) as revealed by anti-calbindin immunohistochemistry (red) and DAPI nuclear staining (blue). Heterozygotes show no detectable alterations in cerebellar size, foliation, or layering. Vermis lobules are labeled III-X; hemispheric lobules are anterior (A), simplex (S), crus I (cI), crus II (cII), paramedian (PM), and pyramidis (P). All photographs taken at the same magnification. Concordant findings from other mutations of the allelic series are shown in Figures S1–S4.

By using gene-driven *N-ethyl-N-nitrosourea* (ENU) mutagenesis screening [5], we generated mice with distinct point mutations in *Foxp2*, backcrossed onto clean genomic backgrounds (Figure 1A; Figure S1 and Supplemental Experimental Procedures available online). The *Foxp2-R552H* line carries an arginine-to-histidine substitution identical to the R553H substitution in affected members of the KE family [1], disrupting DNA-binding and transactivation properties [6]. *Foxp2-N549K* mice harbor a substitution nearby, at another conserved site of the DNA-binding domain. The *Foxp2-S321X* line carries a premature stop codon close to the human R328X nonsense mutation found in a second family segregating *FOXP2*-related speech and language deficits [7]. Although levels of Foxp2 mRNA and protein are normal in R552H and N549K mutants, S321X homozygotes show reduced quantities of *Foxp2* messenger RNA (mRNA) (likely due to nonsense-mediated RNA decay), absence of Foxp2 protein, and no detectable truncated product (Figure S1). Intermediate levels of mRNA and protein are observed for S321X heterozygotes. Thus, S321X is effectively a null allele, implying a similar mechanism for the human R328X mutation [7].

R552H and S321X homozygotes show reduced weight gain, which is not explained by feeding difficulties or lack of maternal care (Figure 1B, Figure S2A). They display delayed maturation of the righting reflex (Figure 1C, Figure S2B), and die approximately 3–4 weeks after birth. The developing lung is a site of Foxp2 expression; complete *Foxp2* loss in knockout mice yields postnatal dilation of distal airspaces, although ultrastructural examinations have found number and morphology of alveolar epithelial cells to be normal [8]. We assessed the hematocrit of R552H and S321X homozygotes (Figure S3). Levels were indistinguishable from wild-type animals, indicating no major oxygenation deficits.

We carried out histological analyses of forebrain structures at P21 in mutants, including conserved Foxp2 expression sites in the cortex, striatum, and thalamus [9, 10], and found no gross abnormalities. However, the cerebellum of homozygotes was disproportionately small, with decreased foliation (Figure 1D; Figure S4). Despite this reduced volume, cerebellar histoarchitecture at P21 remained intact—Purkinje cells (PCs) aligned in a continuous row, and granule cells had completed migration into the internal granular layer (Figure 1D; Figure S4). N549K homozygotes display comparable deficits in postnatal development and cerebellar volume to R552H and S321X homozygotes, but symptoms are milder and show variable expressivity, with prolonged survival (3–5 months).

Consistently across the allelic series, our heterozygous *Foxp2* mutants are fully viable and healthy, without delays in weight gain or in righting-reflex maturation (Figures 1B and 1C, Figure S2). Heterozygote brain development appears grossly normal, even for the cerebellum, where size and histoarchitecture do not differ from wild-types (Figure 1D, Figure S4).

### *Foxp2-R552H* Heterozygotes Display Impaired Motor-Skill Learning

Understanding of the neural bases of *FOXP2*-related disorder in humans comes primarily from investigations of the KE family. Affected family members carry a heterozygous R553H mutation yielding difficulties in sequencing mouth movements, as well as impaired expressive and receptive language [2]. Structural neuroimaging has shown that brains of affected people are overtly normal, but voxel-based morphometry detected subtle changes in gray-matter density, most notably in the inferior frontal gyrus, striatum, and cerebellum [11]. Functional neuroimaging revealed abnormalities during covert (silent) language tasks, including underactivation of the inferior frontal gyrus and putamen [12]. Thus, it has been proposed that this heterozygous R553H mutation affects frontostriatal and/or frontocerebellar networks involved in learning, planning, and execution of rapid movement sequences [4]. Our *Foxp2-R552H* heterozygous mice facilitated the first in vivo investigations of this KE point mutation in an animal model.

**Figure 2:**
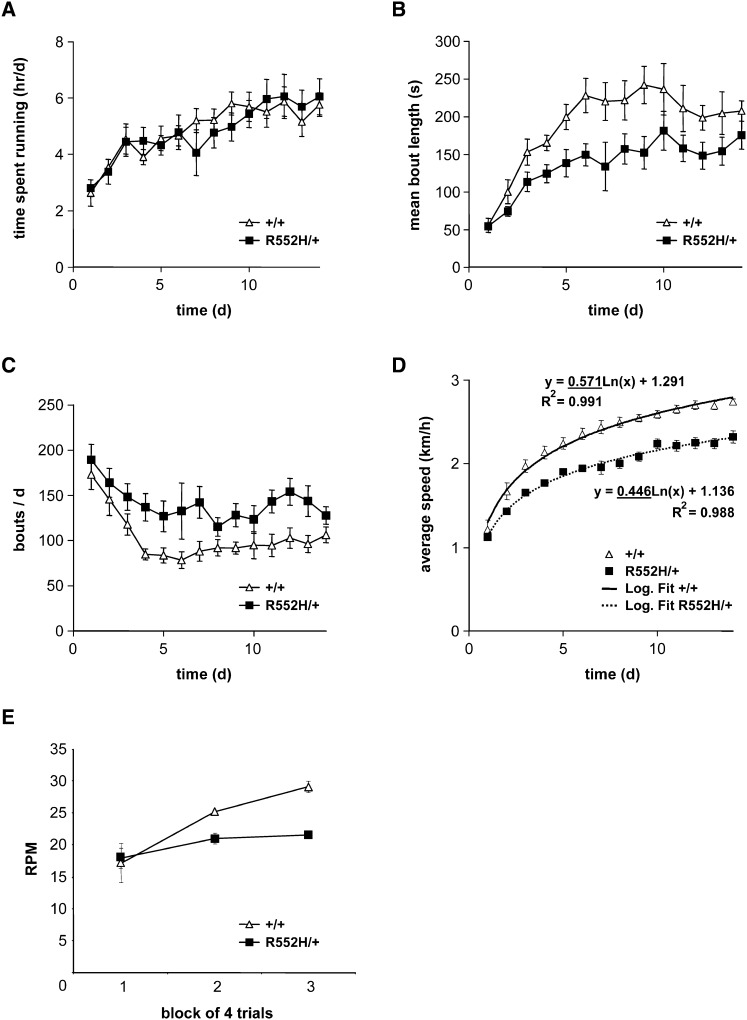
Impaired Motor-Skill Learning of *Foxp2-R552H* Heterozygous Mice on Voluntary Running-Wheel Systems and Accelerating Rotarods (A) Heterozygous R552H mice (n = 9) and wild-type littermates (n = 9) spend the same amount of time running per day (AUC [38], t test, p = 0.95), indicating similar motivation, fitness, and levels of fatigue. (B) Mean bout length per day shows a sharp increase as mice learn to use the wheel. However, heterozygous R552H mice do not learn as rapidly as wild-type littermates, and hence run significantly shorter bout lengths during the 2 week period (AUC, t test, p < 0.05). Note that performance is equivalent on day 1 and that heterozygotes and wild-types converge toward the end of the test period. (C) Mean bout number per day shows a characteristically steep drop after the first day. Although performance is equivalent at the outset, heterozygous R552H mice display a more gradual decrease than do wild-type littermates and thus run significantly increased bout numbers (AUC, t test, p < 0.005). Again, wild-type and heterozygous performance converges toward end of the 2 week period. (D) Heterozygous R552H mice display significantly reduced average speeds (AUC, t test, p < 0.0001). There is no speed difference on day 1, but the subsequent rate of learning is significantly slower in heterozygous mice compared to wild-type littermates (slope of the logarithmic curve fit, p < 0.01). (E) An independent cohort of heterozygous R552H mice (n = 10) display deficits in motor-skill learning compared to wild-types (n = 10) during learning on the accelerating rotarod (analysis of variance [ANOVA] of trials x genotype, p < 0.0001) (RPM indicates revolutions per minute). All panels show mean ± SEM.

R552H heterozygotes show normal embryonic and postnatal development (Figure 1) and base-line motor abilities (Table S1, Figure S5). We assessed motor-skill learning by using a tilted voluntary running-wheel system that enables investigations of species-typical motor patterns in home-cage environments without being confounded by stress responses to handling or disrupted diurnal rhythms [13]. All mice spent a substantial amount of time on the wheels, running in typical short bouts (Figure 2A). After the first day, bout length showed a characteristic rapid increase, accompanied by a steep drop in bout number (Figures 2B and 2C). These rapid early changes in motor patterns reflect behavioral learning rather than alterations in cardiovascular fitness [13]. Importantly, genotype did not affect performance on the first day. However, R552H heterozygotes subsequently displayed slower increases in bout length than wild-type littermates, paralleled by more gradual declines in bout number (Figures 2B and 2C), indicating significant (p < 0.005) deficits in learning to use the wheel. Furthermore, the rate of increase in average running speed, a separate index of motor-skill learning [13], was significantly lower in heterozygotes (p < 0.01; Figure 2D).

Although Foxp2 is expressed in the developing lung, these findings cannot be explained by respiratory impairment. Throughout voluntary training, there was no effect of genotype on time spent running, indicating similar fitness and motivation in R552H heterozygotes and wild-type littermates (Figure 2A). At the outset, heterozygotes showed normal performance—on day 1, average speed, distance, time spent running, bout length, and bout number did not differ from those of wild-types (Figures 2A–2D). Furthermore, although heterozygotes are slower to learn, they do achieve rapid speeds (Figure 2D), and bout length and bout number eventually reach wild-type levels. Finally, in studies of targeted *Foxp2* knockouts (see below), extensive investigations of postnatal lung alveolarization and distal airways did not detect any lung abnormalities in heterozygotes [8].

To further exclude respiratory confounds, we studied independent cohorts of R552H heterozygotes by using accelerating rotarods, an alternative paradigm routinely used for the assessment of rodent motor-skill learning [14]. On the first trials, heterozygotes performed similarly to wild-type littermates, but they subsequently improved at significantly slower rates (p < 0.0001; Figure 2E), mirroring the voluntary running-wheel data. R552H heterozygotes did not show enhanced anxiety in the elevated-plus-maze or open-field arena, altered spontaneous locomotor activity in automated recordings, general cognitive deficits in the T maze, or abnormal grooming (Table S1, Figure S5).

### *Foxp2-R552H* Heterozygotes Show Abnormal Synaptic Plasticity

Previous work highlighted the cortex, striatum and cerebellum (specifically PCs and deep cerebellar nuclei) as key sites of embryonic and postnatal Foxp2 expression [9, 10]. Corticostriatal and corticocerebellar circuits are known to mediate motor-skill learning [15], although their potential distinctive contributions to such abilities remain unclear. Given the absence of overt morphological abnormalities in R552H heterozygous brains, we used electrophysiology to assess functional properties of striatal and cerebellar neurons from these animals.

**Figure 3:**
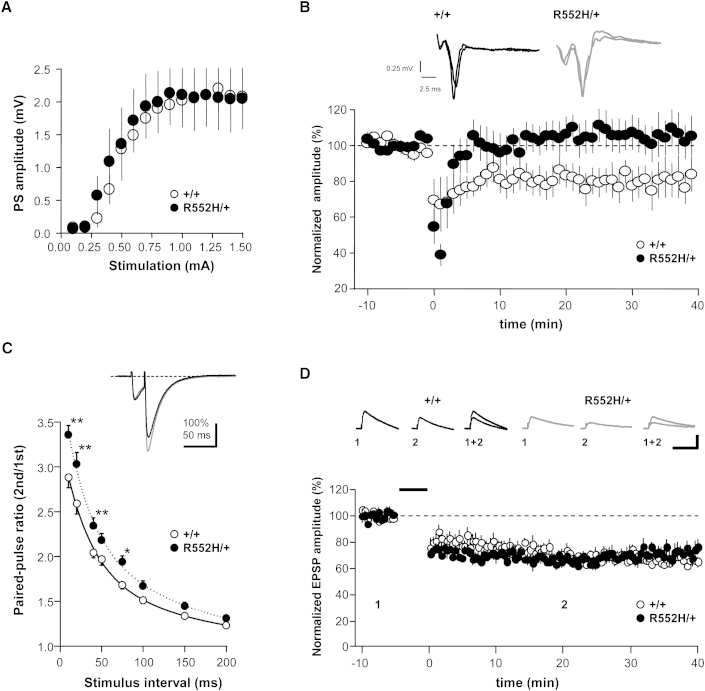
Abnormal Synaptic Plasticity of *Foxp2-R552H* Heterozygous Mice in the Striatum and Cerebellum (A) Input-output functions of evoked potentials in dorsolateral striatum for R552H heterozygous (n = 5) and wild-type (n = 5) mice (mean ± SEM). There was no significant interaction between genotype and function (F_14,70_ = 0.12, p > 0.05). (B) Summary LTD data in dorsolateral striatum for R552H heterozygous (n = 7) and wild-type (n = 6) mice, showing mean amplitudes for every minute (mean ± SEM). Representative traces during baseline and after high frequency stimulation are shown at the top. There was a significant interaction between genotype and LTD (F_55,605_ = 2.62, p < 0.05). Wild-type mice showed significant striatal LTD (post hoc, p < 0.05), whereas R552H heterozygotes did not (post hoc, p > 0.05). (C) Paired-pulse facilitation of PF inputs to cerebellar PCs in R552H heterozygous (n = 16) and wild-type (n = 15) mice show significant differences at short interstimulus intervals (ANOVA, ^∗^ indicates p < 0.05, ^∗∗^ indicates p < 0.01, mean ± SEM). Solid and dashed lines represent double exponential fits to the data. Example traces (average of five EPSCs) for a stimulus interval of 20 ms are shown in the inset. (D) Summary LTD data in PCs from R552H heterozygous (n = 7) and wild-type (n = 12) mice (mean ± SEM). The black bar indicates the induction period (300 combined CF-PF stimulations at 1 Hz). Example traces (average of 12 EPSPs) from indicated time points are shown at the top. Stimulation artifacts were clipped for clarity. Scale bars are 2mV and 50 ms. Significant LTD was induced in PCs of both groups, indicated by paired t tests comparing for each animal the average baseline level with the average value during final 10 min of recording [39] (R552H heterozygotes, df = 6, t = 6.07, p < 0.001; wild-type, df = 11, t = 14.28, p < 0.001). In wild-types, there was a gradual establishment of LTD, with a significant drop in EPSPs between the initial 10 min postinduction and subsequent 10 min windows (t = 2.63–2.87, p < 0.025, df = 11). In contrast, heterozygotes showed more immediate and stable depression of EPSPs (comparison of initial 10 min postinduction to subsequent 10 min windows; t = −0.29–1.28, p > 0.16, df = 6).

For assessment of striatal function, we recorded evoked field potentials from the dorsolateral striatum, a region previously implicated in skill learning on the accelerating rotarod [16–18]. The input-output function—reflecting the amplitude of the evoked population spike in relation to the stimulation strength—was similar between R552H heterozygotes and wild-type littermates (Figure 3A). There were also no differences in the stimulation strength necessary to evoke half of the maximum response (WT = 0.92 ± 0.11 mA, R552H = 0.90 ± 0.13 mA, t_15_ = 0.12, p > 0.05). Thus, R552H heterozygotes do not display major abnormalities in synaptic organization or transmission in the dorsolateral striatum.

We next examined synaptic plasticity in this region, investigating long-term depression (LTD) at glutamatergic synapses induced by high-frequency stimulation, a form of plasticity mediated by endocannabinoid retrograde signaling [19–21]. There were significant differences in striatal synaptic plasticity between wild-type mice and R552H heterozygotes (p < 0.05), with only the former displaying significant LTD (Figure 3B). In contrast, R552H heterozygotes showed depression of the evoked responses immediately after high-frequency stimulation, but no LTD (p > 0.05). These data indicate that LTD in the dorsolateral striatum of R552H heterozygotes is strongly impaired.

For examination of cerebellar circuits, we employed electrophysiological recordings and Ca^2+^ imaging to assess climbing-fiber (CF) and parallel-fiber (PF) inputs of PCs in acute slices. CF fibers undergo a characteristic elimination of redundant inputs during the first two postnatal weeks. Disturbed cerebellar development typically yields multiple weak CF inputs that fail to form strong one-to-one multisite synaptic contacts with the PC [22]. CF-induced inputs in R552H heterozygotes were normal, with no indication of disturbed CF elimination (Figures S6A–S6C). Regardless of genotype, only about 10% of PCs showed multiple CF innervation, typical for normal development [22]. Furthermore, CF stimulation triggered full-blown dendritic Ca^2+^ transients with peak amplitudes and temporal integrals indiscernible from those of wild-types (Figures S6A and S6B). Similarly, activation of PF inputs yielded normal excitatory postsynaptic potentials mediated by AMPA-type glutamate receptors. Therefore, R552H heterozygotes display a grossly normal cerebellar synaptic circuitry.

We went on to assess two types of cerebellar synaptic plasticity. (1) Paired-pulse facilitation of the PF-PC synapse represents a form of short-term plasticity resulting primarily from enhanced presynaptic transmitter release due to residual Ca^2+^ ions [23] and partly from extracellular K^+^ accumulation [24]. Paired-pulse facilitation in R552H heterozygotes was significantly (p < 0.01) enhanced at short interstimulus intervals (10–75 msec) as compared to that in wild-type littermates, with no detectable differences between genotypes at longer intervals (Figure 3C). (2) LTD at PF-PC synapses has been proposed as a cellular substrate of motor-skill learning in the cerebellum [25]. Simultaneous PF-CF activation induced robust LTD of similar magnitude in PCs of both groups. However, we detected subtle differences in the temporal profile in R552H heterozygotes as compared to wild-types, consistent with faster induction of LTD in the former (Figure 3D).

### Vocalizations of *Foxp2-R552H* Mutants

Finally, prompted by a recent study [26], we investigated innate vocalizations of R552H mutants. Rodents produce three sound types, broadband audible, pure ultrasonic, and broadband click sounds, that differ substantially in physical structure and production mechanisms. In particular, although audible sounds are produced by the vibration of vocal cords, ultrasounds appear to be generated by expiration through a small opening between arytenoid cartilages with otherwise tightly opposed, nonvibrating vocal cords (like a whistle) [27, 28]. Thus, ultrasound emission is likely to require more muscle power due to greater airway resistance.

**Figure 4:**
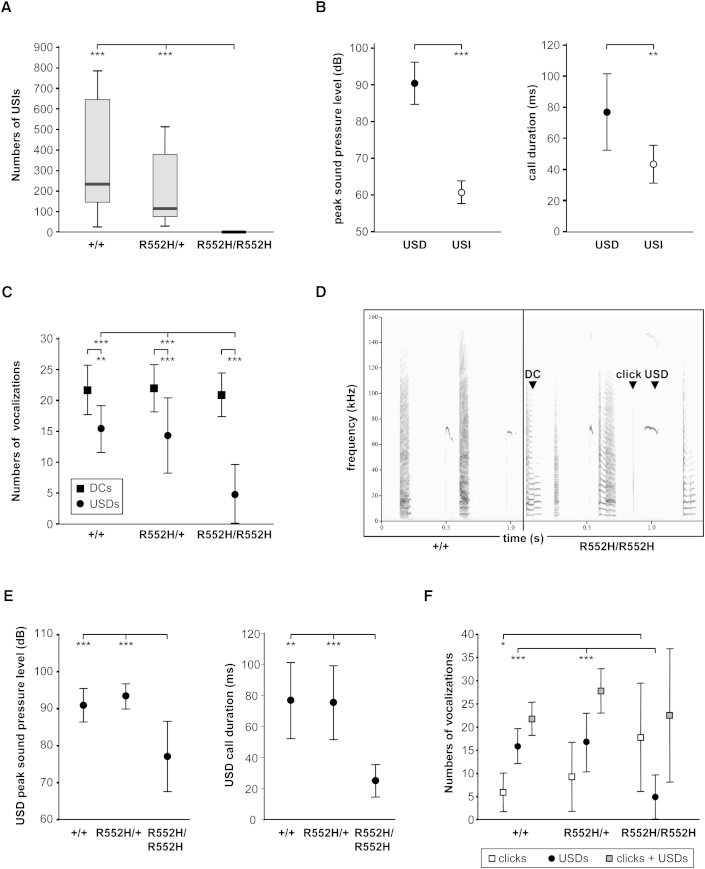
Vocalizations of Heterozygous and Homozygous R552H Pups (A) Number of USIs produced during 15 min recording. Significant differences exist between wild-type (n = 9) and R552H homozygous (n = 8) and between heterozygous (n = 10) and homozygous mice (ANOVA on ranks, p < 0.001; U test, p < 0.001). There are no significant differences between wild-types and heterozygotes (U test, p = 0.236). (B) Properties of USDs and USIs in wild-type mice. Peak sound pressure (left) differs between USDs and USIs in wild-types (n = 9; t test, p < 0.001). Call duration (right) differs between USDs and USIs in wild-types (n = 8 for USIs, n = 9 for USDs; t test, p < 0.01). (C) Number of DCs and USDs in initial 10 s of recording. Number of DCs does not differ between groups (one-way ANOVA, p > 0.05). Homozygotes (n = 8) produce fewer USDs than do heterozygotes (n = 16) and wild-types (n = 9) (one-way ANOVA, p < 0.001; Tukey test, p < 0.001). All groups emit more DCs than USDs (t test, at least p < 0.01). (D) Sample sonograms of DCs interspersed with USDs and clicks for wild-type and R552H homozygous pups. (E) Peak sound pressure of USDs (left) in R552H homozygotes (n = 7) is significantly lower than heterozygotes (n = 10) and wild-types (n = 9) (one-way ANOVA, p < 0.001; Tukey test, p < 0.001). Call duration of USDs (right) is significantly reduced in homozygotes (n = 5) as compared to heterozygotes (n = 16) (Tukey test, p < 0.001) and wild-types (n = 9) (one-way ANOVA, p < 0.001; Tukey test, at least p < 0.01). (F) Number of USDs and clicks in first 10 s of recording. R552H homozygotes (n = 8) emit significantly more clicks than do wild-types (n = 8) (one-way ANOVA, p < 0.05; Tukey test, p < 0.05) and significantly fewer USDs than do wild-types (n = 8) and heterozygotes (n = 10 for clicks and USDs, n = 9 for sum of clicks and USDs) (one-way ANOVA, p < 0.001; Tukey test, p < 0.001). The sum of clicks and USDs does not differ between groups (one-way ANOVA, p > 0.05). In all panels, ^∗^ indicates p < 0.05, ^∗∗^ indicates p < 0.01, and ^∗∗∗^ indicates p < 0.001. In (B), (C), (E), and (F), mean ± standard deviation (SD) is shown.

We initially studied isolation calls. Young pups when isolated from the nest emit frequency-modulated tones in the high ultrasonic range (here called USIs), accompanied by clicks [29]. When comparing R552H heterozygotes to wild-type littermates, we found no significant difference in numbers of USIs produced (Figure 4A) or in their general characteristics (e.g., call duration, peak sound-pressure level, minimum and maximum frequency, and mean number of calls in a series). However, R552H homozygotes did not emit any USIs. Importantly, USIs elicit a phonotaxic approach in adults, a response resembling that of humans toward crying babies. Pup ultrasounds reflect a modulated arousal state yielding altered motor activity, including ultrasound production, rather than a motivation to acoustically communicate [30, 31]. Thus, the absence of homozygous USIs could potentially result from reduced arousal in isolation as compared to wild-type and heterozygous animals.

Therefore, we next employed situations yielding higher arousal and stronger efforts to vocalize. When newborns are lifted a short distance above the ground (Supplemental Experimental Procedures), they emit audible distress calls (DCs) with interspersed ultrasounds (here called USDs) and clicks. We compared USDs to USIs in wild-type mice, finding that USDs are louder (higher peak sound-pressure level) and longer (Figure 4B), consistent with greater arousal in the distress condition. R552H homozygotes, heterozygotes, and wild-type littermates emitted equivalent numbers of DCs (Figure 4C), indicating similar arousal in the different groups. All mice also produced USDs (Figure 4C). Thus, in these conditions, R552H homozygotes generate both harmonically structured audible calls and complex ultrasonic whistles, suggesting that neural mechanisms of motor coordination for ultrasound production are intact (Figure 4D).

Nevertheless, R552H homozygotes emitted significantly fewer USDs than did wild-type and heterozygous littermates (Figure 4C). A parsimonious interpretation is that the difficulties of homozygotes in making ultrasounds might be secondary to their severe developmental delays, gross motor difficulties, and/or reduced physical well being. USDs of homozygotes have lower sound-pressure levels and shorter duration than those of heterozygotes and wild-types (Figure 4E). Moreover, R552H homozygotes emit significantly more clicks under distress than do wild-types (Figure 4F). Clicks often precede USDs and so might indicate the intention of mice to produce USDs. Indeed, if numbers of clicks and USDs are summed, the total number of produced sounds is similar in all groups (Figure 4F), concordant with R552H homozygotes' being equivalently aroused under distress but physically less able to produce ultrasounds.

### Comparison to Targeted Knockouts

Previously, Shu and colleagues used standard gene-targeting to replace exons 12–13 of *Foxp2* with a *Neomycin* selection cassette [26], yielding knockout mice (referred to here as *Foxp2-KO*s). Our homozygous mutants show overt similarities to the described *Foxp2-KO* homozygotes, including reduced weight-gain, delayed righting-reflex maturation, and postnatal lethality. However, Shu et al. reported abnormalities in cerebellar histoarchitecture in *Foxp2-KO* homozygotes at P15–17; PCs frequently failed to align in a monolayer, and there was continued presence of an external granular layer (EGL) [26]. These observations might reflect an earlier state of cerebellar maturation associated with severe developmental delays. Detailed examination of our various homozygous mutants at P21 indicates a cerebellum with reduced volume but well-preserved histoarchitecture, including normal PC alignment and no persistent EGL (Figure 1D, Figure S4). Moreover, Shu and colleagues emphasized the complete absence of ultrasonic isolation calls made by *Foxp2-KO* homozygotes [26]. We similarly find no such calls in *Foxp2-R552H* homozygotes but demonstrate that these mice do produce complex structured ultrasounds in situations involving higher arousal (Figure 4) and suggest that their vocalization problems could be secondary to physical difficulties.

The heterozygous mutants of the present study also display notable differences from *Foxp2-KO* heterozygotes, which were reported to show moderate developmental delays and abnormal cerebellar histoarchitecture, including retainment of a one-cell-thick EGL at P17 [26]. Our heterozygotes, consistently across the allelic series, are viable and healthy, with normal postnatal weight gain, righting-reflex maturation, baseline motor skills, locomotor activity, and cerebellar size and morphology. In addition, although *Foxp2-KO* heterozygotes apparently display significantly reduced ultrasound production, with normal call properties [26], we find that *Foxp2-R552H* heterozygotes produce similar numbers of ultrasounds (whether USIs or USDs) as do wild-type littermates. Rates of ultrasound production follow characteristic strain-specific ontogenetic profiles, closely related to postnatal development [32]. Thus, the reduced USI production seen in *Foxp2-KO* heterozygotes, but not our *Foxp2-R552H* heterozygotes, might reflect the fact that only the former display developmental delays.

Overall, our data demonstrate the importance of considering arousal state, developmental delay, and physical well being when one is studying innately-specified vocalizations of mutant/knockout mice. Clearly, the relationship between *Foxp2* damage and vocalization changes is more complex than previously proposed [26].

### Significance of *Foxp2-R552H* Mice

There is an emerging consensus that distributed circuits involving the cortex, basal ganglia, and cerebellum play key roles in mediating both motoric and cognitive function [33, 34]. With regard to *FOXP2* and speech and language disorders, it has been hypothesized that etiological mutations in this gene affect corticostriatal and corticocerebellar networks that facilitate the learning and production of rapid movement sequences [4] and that might also be important for aspects of language function [33]. Our study represents the first assessment of motor-skill learning and functional properties of relevant circuits in mice carrying *Foxp2* disruptions and focuses on a point mutation whose effects have been particularly well studied in humans. We find that *Foxp2-R552H* heterozygous mice display subtle but highly significant deficits in learning of rapid motor skills during species-typical behaviors. Moreover, we uncover abnormalities in synaptic plasticity in two key sites of conserved Foxp2 expression with established links to motor-skill learning; striatal neurons and cerebellar PCs. These data are consistent with proposals that human speech faculties recruit evolutionarily ancient neural circuits involved in motor learning [33].

The complete lack of LTD in the dorsolateral striatum (putamen) of heterozygous *Foxp2-R552H* mice is intriguing, given that putamen underactivation was a major finding from functional neuroimaging of heterozygous *FOXP2-R553H* humans [12]. Delineation of the precise in vivo mechanisms that yield this disturbed synaptic plasticity in mutant mice promises to shed important new light on our understanding of the disorder. Furthermore, our data support recent studies of songbird *FoxP2* that similarly indicate that the gene's effects on cognition and behavior go beyond development, to involve “online” roles in striatal neurons [35, 36]. Conditional mice in which *Foxp2* can be inactivated in a spatially and temporally selective manner [37] should allow separation of developmental and online functions. Such mice will also be essential for dissecting out relative contributions of striatal and cerebellar dysfunction to motor-skill learning deficits associated with *Foxp2* disruption.

## Supplemental Data

Document S1. Experimental Procedures, Seven Figures, and One Table
