# Supplementary material for: Impaired Synaptic Plasticity and Motor Learning in Mice with a Point Mutation Implicated in Human Speech Deficits
Source: Curr Biol. 2008 Mar 11;18(5):354–62. doi: 10.1016/j.cub.2008.01.060 (PMC2917768; doi:10.1016/j.cub.2008.01.060)
Supplement: Document S1. Experimental Procedures, Seven Figures, and One Table [file mmc1.pdf]

Impaired Synaptic Plasticity and Motor Learning in Mice with a Point Mutation Implicated in Human Speech Deficits

Matthias Groszer, David A. Keays, Robert M.J. Deacon, Joseph P. de Bono, Shweta Prasad-Mulcare, Simone Gaub, Muriel G. Baum, Catherine A. French, Jérôme Nicod, Julie A. Coventry, Wolfgang Enard, Martin Fray, Steve D.M. Brown, Patrick M. Nolan, Svante Pääbo, Keith M. Channon, Rui M. Costa, Jens Eilers, Günter Ehret, J. Nicholas P. Rawlins, and Simon E. Fisher

Supplemental Experimental Procedures

Animals

Generation of Foxp2 Mutant Mice

We used a gene-driven ENU-based screening approach to generate an allelic series for murine *Foxp2*. R552H and N549K mice were derived from a large scale ENU mutagenesis project at Medical Research Council Harwell laboratories (Oxfordshire), United Kingdom [S1]. ENU was injected in founder mice of a BALB/c genetic background. DNA from approximately 5,400 F1 offspring was obtained. The region encoding the forkhead domain of Foxp2, comprising exons 12, 13, and 14, was amplified in all samples by polymerase chain reaction (PCR) with the following primers: Foxp2_12/13_F: 5′-CCAGTGGTTTCTCACCAAATC-3′, Foxp2_12/13_R: 5′-CAGGAGGACTTTGGCGTTAC-3′, Foxp2_14_F: 5′-GCTCCTCTTGGCTCTTTACCG-3′, and Foxp2_14_R: 5′-TGTGAGCATGCCTTTAGCTG-3′.

PCR conditions were as follows: 95°C for 15 min (1 cycle), 95°C for 30 s, 62°C for 30 s at −0.5°C/cycle, 72°C for 60 s (13 cycles), 95°C for 30 s, 55°C for 30 s, 72°C for 60 s (29 cycles), and 72°C for 7 min (1 cycle). Amplified products were screened for mutations with the WAVE DNA Fragment Analysis System (Transgenomic, Cheshire, United Kingdom). DNA samples with abnormal elution profiles were amplified and sequenced. Identified mutations R552H and N549K were derived from two different ENU mutagenized founder males.

S321X mice were derived from commercial parallel archives of genomic DNA and frozen sperm from approximately 17,000 F1 offspring of male C3H mice treated with ENU at Ingenium Pharmaceuticals, Germany as described [S2].

ENU mutagenesis yields multiple mutations at random throughout the genome [S3]. Nevertheless, it is highly unlikely that the observed phenotypes are explained by ENU-induced mutations other than those in *Foxp2*. Prior to phenotypic characterization, we employed marker-assisted backcrossing as described [S4] to eradicate potential confounding mutations from the genomic background (see below). On the basis of microsatellite marker analyses, we estimate that the probability of finding more than one functional mutation in our backcrossed animals is 0.0404 for the R552H line and 0.0526 for S321X. Moreover, the different *Foxp2* alleles provided concordant phenotypic findings, even though they were isolated from two independent ENU archives [S1, S2], each line being derived from a separate founder male. Further support comes from allelic intercrosses, with R552H/S321X compound-heterozygotes showing identical phenotypes to those of pure R552H or S321X homozygotes, and there was phenotypic consistency across two alternative genomic backgrounds (C57BL/6J and C3H).

Marker-Assisted Backcrossing

F1 founder males were backcrossed into the C57BL/6J and C3H backgrounds. To accelerate introgression of the *Foxp2* mutant alleles, and to eliminate flanking heterozygous DNA, we conducted marker-assisted backcrossing. After each backcross, microsatellites with known polymorphic differences on chromosome 6 were amplified from genomic DNA. For backcross C3H, the following markers were employed: D6mit138, D6mit204, D6mit116, D6mit223, D6mit384, and D6mit243. For backcross C57BL/6J, the following markers were employed: D6mit138, D6mit166, D6mit83, D6mit1, D6mit264, D6mit204, D6mit159, D6mit223, D6mit245, and D6mit70. PCR reactions were conducted in a total volume of 10 μl (2 μl of 5 ng/μl DNA, 1 μl 10x PCR buffer, 1 μl dNTPs [8 mM], 0.6 μl $MgCl_2$ [25 mM], 1 μl primers [10 μm], 0.05 μl Taq gold, and 4.35 μl H_2O), under the following conditions: 95°C for 15 min (1 cycle), 95°C for 30 s, annealing temperature for 30 s, 72°C for 30 s (30 cycles), and 72°C for 5 min (1 cycle). Mice with the smallest heterozygous region carrying the mutation were selected for backcrossing.

Genotyping

R552H and N549K mice were genotyped by PCR and restriction digestion of genomic DNA. The following primers were used: *Foxp2-R552H* forward: 5′-GTTCCTCTGGACATTTCAAC-3′ and *Foxp2-R552H* reverse: 5′-TGTGAGCATGCCTTTAGCTG-3′. PCR conditions were as follows: 94°C for 1 min (1 cycle), 94°C for 30 s, 55°C for 30 s, 68°C for 1 min (35 cycles), and 72°C for 10 min (1 cycle). For R552H mice, the 603 bp PCR products were digested with HgaI yielding fragments of 372 bp and 231 bp in wild-type mice but remained undigested in R552H homozygous mutant mice. For N549K mice, the PCR products were digested with BsmI yielding fragments of 380 bp and 223 bp in wild-type mice but remained undigested in N549K homozygous mutant mice.

S321X mutant mice were genotyped by PCR of genomic DNA with the following primers: *Foxp2-S321X* forward: 5′-ATAGTATGGAAGACAACGGCATC-3′ and *Foxp2-S321X* reverse: 5′- GATGGGGTTAGTGAATGTTCTCA-3′. PCR conditions were as follows: 95°C for 15 min (1cycle), 94°C for 1 min, 55°C for 1 min, 72°C for 1 min (35 cycles), and 72°C for 10 min (1cycle). The 468 bp PCR products were digested with AflII, yielding fragments of 332 bp and 136 bp in S321X homozygous mutant mice; DNA of wild-type mice remained undigested.

Maintenance

Mice were maintained on a 12 hr light/dark cycle at a temperature of 22°C, 60%–70% humidity, with ad libitum food and water access. Cages were provided with cardboard tunnels and wood sticks for environmental enrichment. Experiments were performed in accordance with the United Kingdom Animals (Scientific Procedures) Act 1986.

Histology

Animals were perfused with ice-cold 4% paraformaldehyde, and brains were removed and processed according to standard procedures. Immunohistochemistry was performed on 5 μm Paraffin sections. After antigen retrieval, sections were incubated with calbindin-D28k (1:500, cat. # CB-38a, Swant, Bellinzona, Switzerland) and GABA-A receptor alpha6 (1:50, cat.# sc-7359, Santa Cruz, USA) antibodies. Tbr1 (rabbit, 1:2000, R. Hevner laboratory, Washington) immunohistochemistry was performed on 40 μm cryosections.

Analyses of Gene and Protein Expression

Western blotting and quantitative real-time reverse transcriptase (RT)-PCR were carried out as described previously [S5].

Striatal Electrophysiology

Brain slices from 3- to 6-month-old R552H heterozygous mice and wild-type littermates containing both striatum and cortex were prepared as described previously [S6]. Animals were anaesthetized with halothane and their brains extracted and transferred rapidly to artifical cerebrospinal fluid (aCSF), which was brought to pH 7.4 by aeration with 95% O_2 and 5% CO_2. Coronal sections (350 μm thick) were cut in ice-cold aCSF with a Vibrotome 1000 slicer (St. Louis, Missouri). Slices were transferred immediately to a nylon net submerged in normal aCSF containing 124 mM NaCl, 4.5 mM KCl, 2 mM $CaCl_2$, 1 mM $MgCl_2$, 26 mM $NaHCO_3$, 1.2 mM NaH_2PO4, and 10 mM d-glucose. Normal aCSF was maintained at pH 7.4 by bubbling with 95% O_2 and 5% CO_2 at room temperature (19°C –22°C). After at least 1 hr of incubation at room temperature, hemislices were transferred to a recording chamber and submerged in aCSF. For all experiments, the temperature of the bath was maintained at 26°C –28°C stable within ± 1°C during any given experiment. Extracellular field recordings were obtained with micropipettes (2–4 MΩ) filled with 1 M NaCl solution. Test stimuli were delivered via a S45 stimulator (Grass Instruments, West Warwick, Rhode Island) at a frequency of 0.05 Hz through a bipolar twisted Teflon-coated tungsten wire placed in the dorsolateral striatum, and population spikes (PS) were recorded. Stimulus intensity was set to yield an evoked PS amplitude approximately half the size of the maximal evoked response. For the induction of long-term

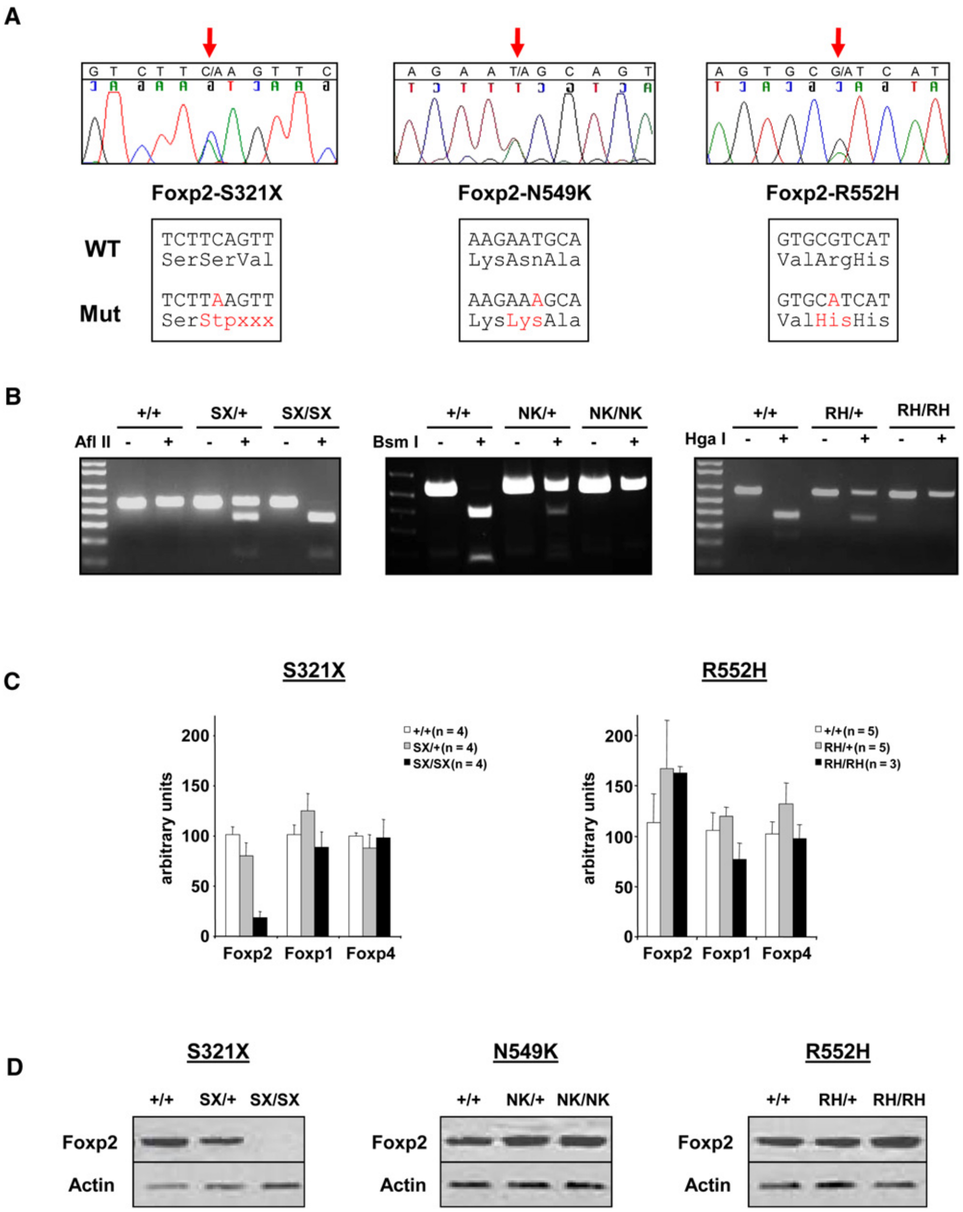

Figure S1. An Allelic Series of *Foxp2* Mutant Mice

(A) Sequence traces showing each heterozygous mutation with corresponding changes in coding sequence below.

(B) Identified mutations change the restriction digest pattern of genomic DNA. S321X introduces an AflII restriction site, and N549K and R552H mutations abolish BmaI and HgaI sites, respectively.

(C) Quantitative RT-PCR from E16 striatal precursor region, showing a significant reduction of *Foxp2* mRNA in S321X homozygotes (t test, WT versus S321X/S321X $p < 0.003$, WT versus S321X/+ $p = 0.19$, mean ± SE). In R552H homozygotes, *Foxp2* mRNA levels are indistinguishable from wild-type littermates. Note that *Foxp2* deficiency does not appear to impact the expression of other *Foxp* family members, *Foxp1* and *Foxp4* in this region.

(D) Western-blot analysis of Foxp2 protein expression in E16 whole cerebellum in *Foxp2* mutant strains, with antibodies recognizing the C terminus of the protein (Serotec). Equivalent results were also obtained when N-terminal antibodies (Santa Cruz) were used.

depression (LTD), four trains of high-frequency stimulation at 100 Hz for 1 s were delivered 10 s apart at the maximal evoked response. Data were filtered (high pass, 0.1 Hz; low pass 3 kHz) and then amplified and digitized with an Axoclamp 1D amplifier and Digidata 1322 interface (Axon Instruments and Molecular Devices, Sunnyvale, California). Input-output data and LTD data were analyzed with two-factor repeated-measures ANOVA, and post hoc unpaired test (PLSD) followed in the case of significant interaction. Stimulating strength necessary to evoke half of the maximum response was analyzed with unpaired t tests.

Cerebellar Electrophysiology

Preparation of Brain Slices

Acute cerebellar slices were obtained from 24- to 28-day-old C57BL/6J R552H heterozygous mice and wild-type littermates as described previously [S7]. The mice were decapitated under isoflurane anesthesia, the cerebellar vermis rapidly removed, and placed in ice-cold aCSF consisting of 125 mM NaCl, 2.5 mM KCl, 2 mM $CaCl_2$, 1 mM $MgCl_2$, 1.25 mM NaH_2PO_4, 26 mM $NaHCO_3$, and 20 mM glucose, bubbled with 95% O_2 and 5% CO_2 (pH 7.4 at room temperature). Parasagittal slices (200 μm thick) were cut

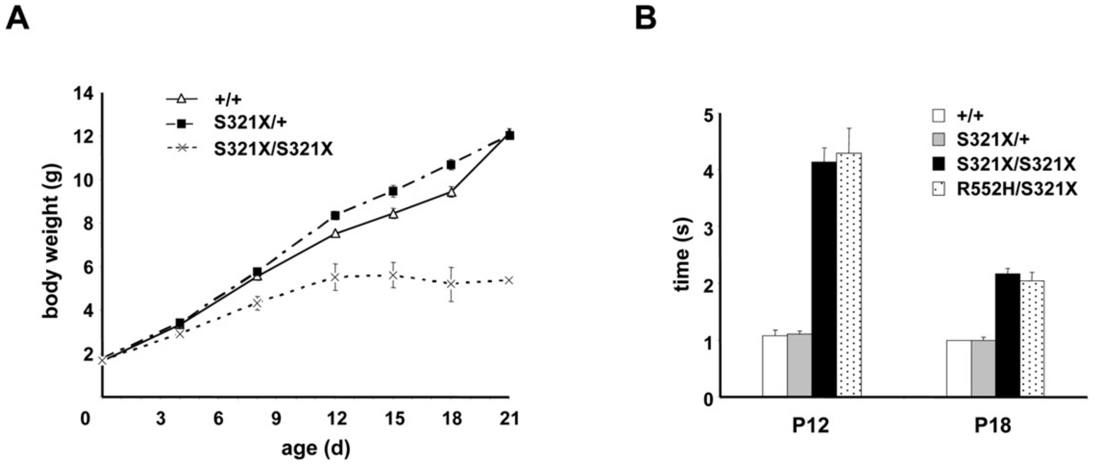

Figure S2. Postnatal Development of *Foxp2-S321X* Mutant Mice

(A) Time course of postnatal body weight development. Homozygous S321X mice (n = 8) show a strongly reduced weight gain. In contrast, S321X heterozygotes (n = 16) are indistinguishable from their wild-type littermates (n = 9) (mean ± SEM). Removal of all S321X heterozygous and wild-type littermates from the cage at postnatal day 4 does not improve weight gain or survival of S321X homozygotes. Therefore, their delays are not simply explained by difficulties in competing for milk and maternal care.

(B) Postnatal righting-reflex development. S321X (n = 6) homozygotes and R552H/S321X compound heterozygotes (n = 6) display a significantly delayed righting reflex. Heterozygous S321X (n = 7) mice are indistinguishable from their wild-type (n = 4) littermates (t test, WT versus S321X/+ p = 0.9, WT versus S321X/S321X p < 0.001).

with a vibratome (Microm HM 650 V, Wolldorf, Germany) and incubated in aCSF at 35°C for 30 to 45 min before they were stored at room temperature (20°C–22°C) until use. All recordings were performed at room temperature and in the presence of 10 μM bicuculline-methiodide (Sigma).

Electrophysiological Recordings

Whole-cell recordings were obtained from the somata of visually identified Purkinje cells (PCs) with an EPC-10 patch-clamp amplifier (HEKA, Lambrecht, Germany) controlled by Patchmaster software (HEKA). Patch pipettes were pulled from borosilicate glass (Hilgenberg, Malsfeld, Germany) to reach 4–6 MΩ resistance when filled with the intracellular solution that contained 150 mM KGlu, 10 mM NaCl, 10 mM HEPES, 3 mM Mg-ATP, 0.3 mM GTP, and 0.05 mM ethylene glycol tetraacetic acid (EGTA) (pH 7.3, adjusted with HCl). Afferent climbing and parallel fibers (CFs and PFs, respectively) were stimulated with a glass pipette placed in the molecular and granular cell layer [S8]. CF inputs were identified by their typical paired-pulse depression and all-or-none characteristics [S8]. Stimulus-response curves of CF inputs and paired-pulse facilitation of PF responses were recorded in the voltage-clamp mode. To allow for a proper voltage clamp, we performed the former recordings in the presence of a submaximal concentration of the AMPA- (α-amino-3-hydroxy-5-methyl-4-isoxazole propionic acid) receptor blocker CNQX (6-cyano-7-nitroquinoxaline-2,3-dione, 1–2 μM) [S8] and with a series resistance compensation of 60%–70%. Imaging and LTD experiments were performed in the current-clamp:bridge mode. For LTD experiments, PF responses were evoked at 0.2 Hz. After a stable baseline of at least 5 min was obtained, LTD was induced by coactivation of CF and PFs at a frequency of 1 Hz for 5 min [S9]. Afterwards, PF stimulation was resumed at 0.2 Hz and LTD induction was monitored for 40 min.

Ca^{2+} Imaging

For analysis of CF-evoked Ca^{2+} transients in dendrites of PCs, EGTA in the intracellular solution was replaced by the Ca^{2+} indicator dye Oregon-Green 488 BAPTA-1 (OGB-1, 200μM). For confocal fluorescence recordings, PCs were dialyzed with the dye containing for 30–45 min before CF-evoked Ca^{2+} signals were recorded in the line-scan mode. Fluorescence signals were background corrected and divided by the baseline fluorescence to yield semiquantitative ΔF/F data. For analysis, the Ca^{2+} transients were fitted with a double exponential function with Igor Pro (Wavemetrics). The amplitudes and the temporal integrals of the Ca^{2+} transients were computed from the fits. Statistical tests were performed with Sigma Stat Software (RockWare).

Behavioral Studies

Righting

In the C57BL/6J strain, the first day of the mature righting response is expected to appear around postnatal day 11 [S10]. Righting was tested by placement of a newborn mouse on its back and the time it took to turn over and place all four paws on the surface was measured. Each animal received four trials, and the mean time was taken.

Tilted Running Wheel

A detailed description of this system has been published recently [S11]. In brief, animals were provided in the home cage with an angled rotating running track (Lillico, Surry, United Kingdom), circumference 37.8 cm, mounted on a greased steel axle (Figure S7). The angled running track was used in preference to traditional vertical wheels so that larger diameter wheels could be used within cages. Wheel running was monitored with a magnetic reed switch attached to a computerized exercise-monitoring system [S11]. This system consisted of a micro 1401 (Cambridge Electronic Design [CED], Cambridge, United Kingdom) capable of simultaneously monitoring in real time the individual rotations of the wheel from up to 44 cages, with wheel rotations recorded and analyzed by computer with Spike 2 software (CED, Cambridge, United Kingdom). For each 24 hr period, the following parameters were recorded: time spent running, average speed of running, and the number and length of individual running bouts. A running bout was defined as episodes of continuous rotation of the wheel with no gaps of greater than 5 s.

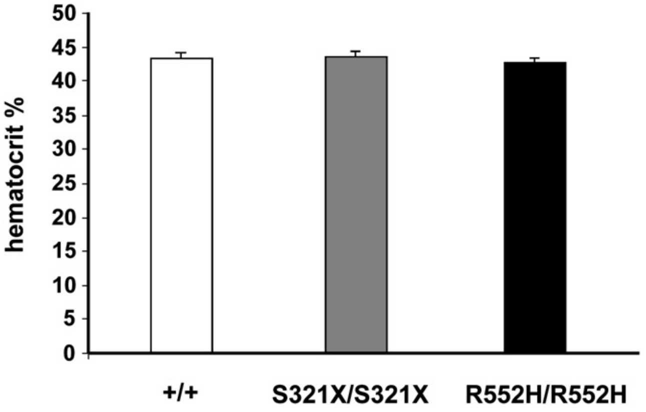

Figure S3. Hematocrit Measurements in *Foxp2* Mutant Mice

S321X and R552H homozygotes show no significant differences from wild-type littermates (n = 3 / group), (mean ± SEM).

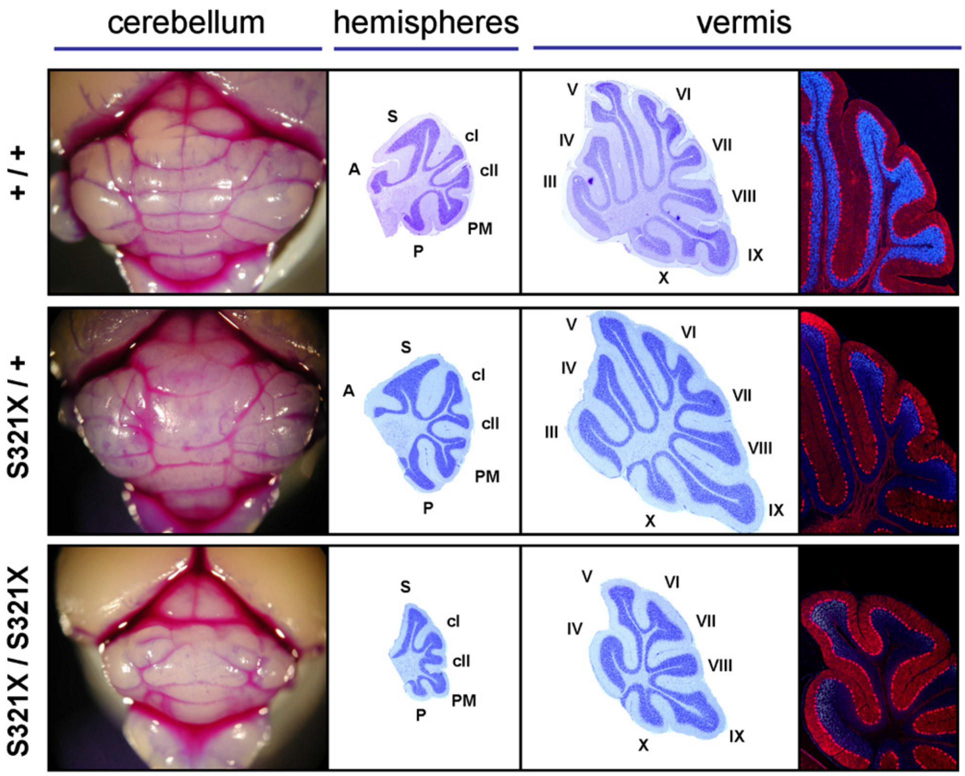

Figure S4. Cerebellar Development of *Foxp2-S321X* Mutant Mice

Cerebellar morphology at postnatal day 21 in wild-type (top row), heterozygous (middle), and homozygous S321X mice (bottom). Homozygotes display reduced cerebellar size (left-hand column) and foliation deficits in hemispheres and vermis (middle columns, cresyl violet staining). Nevertheless, Purkinje cells are aligned in a monolayer (right-hand column) as revealed by anti-calbindin immunohistochemistry (red) and DAPI nuclear staining (blue). Heterozygotes show no detectable alterations in cerebellar size, foliation, or layering. Vermis lobules are labeled III-X; hemispheric lobules are anterior (A), simplex (S), crus I (cI), crus II (cII), paramedian (PM), and pyramidis (P). All photographs were taken at same magnification. R552H/S321X compound heterozygotes show a similar cerebellar phenotype to the homozygotes, again involving reduced size/foliation but preserved architecture (data not shown).

Accelerating Rotarod

Mice were given four trials per day (1 hr intertrial interval) for three consecutive days. Rotarod studies were performed on an apparatus as described previously [S12]. Animals were matched for sex and litter and were between 6 and 8 weeks of age. Average weights (g ± SD) for these animals were as follows: Males: wild-type (n = 5), 22.21 ± 1.15; R552H heterozygotes (n = 5), 23.13 ± 0.94 (p = 0.16), Females: wild-type (n = 5) 18.2 ± 1.0; and R552H heterozygotes (n = 5): 17.5 ± 1.4 (p = 0.4).

Open Field

This employed a brightly lit circular arena with a diameter of 60 cm. Mice were placed in the periphery of the open field and assessed for 5 min by a video camera mounted on the top of the open field. A Videotrack (version NT4.0) automated tracking system from Viewpoint (Champagne Au Mont D'or, France) was used for analysis of the video data.

Elevated Plus Maze

The elevated plus maze consisted of two opposing enclosed arms (30 × 6 × 21 cm), two opposite open arms (29.5 × 6 × 0.5 cm), and a central area (14.5 × 14 cm). The apparatus was elevated 73 cm from the floor. Mice were placed in the center of the plus maze at the beginning of the test, and movements were monitored for 5 min with a mounted video camera. Video data were analyzed with the Videotrack system (Viewpoint).

Locomotor Activity

Basal locomotor activity was assessed with two independent systems: (1) With the San Diego Instruments Photoactivity system, which involves placement of each mouse individually in a plastic cage that has seven infrared beams crossing the width of the cage floor (46 cm × 15 cm × 21 cm). A thin layer of clean wood chips was placed in the cage, and the number of beam breaks measured in 5 min bins over a 30 min period. (2) In individual cages similar to the home cage, placed on load platforms of a Med Associates Threshold activity system. Each cage contained a measured volume of wood-chip bedding creating a layer approximately 0.5 cm thick; this ensured equal foot force damping in all cages. Sessions lasted 1 hr, divided into 12 intervals (bins) of 5 min each. The lower threshold of the system was set to 20 V, and the parameter measured was the number of times this threshold was crossed.

T Maze Spontaneous Alternation

A mouse was placed in the start arm of a gray wooden T maze and allowed to choose one of the goal arms, in which it was confined for 30 s. It was then replaced in the start arm and allowed to make a second choice. An alternation was defined as the mouse's choosing the opposite arm to that entered on the previous, sample run. Mice were given one or two trials/day for a total of five trials each [S13].

Grooming

Mice were placed individually into gray wooden boxes (27 × 9 × 30 cm) with 1 cm of wood-chip bedding from the home cage on the floor. After a habituation period of 30 s, they were observed for 5 min. The number and total duration of grooming bouts was measured by an event counter and timer.

Vocalization Studies

So that the influence of the developmental delay in R552H homozygous mice on our vocalization studies could be reduced, newborn mice were studied at postnatal day 4, when peripheral thermoregulation is still immature in wild-type animals, and body weights were not significantly different between all groups (g ± SD, WT: 2.66 ± 0.52; R552H/+: 2.73 ± 0.48; and R552H/R552H: 2.23 ± 0.49; t test: WT versus R552H/R552H, p = 0.12). Vocalizations were recorded in a soundproof and anechoic room with dim red light at an average temperature of 23°C. For recording of isolation calls (USIs), a newborn was separated from its mother and placed in a dish (diameter: 14 cm, height: 4.5 cm). USIs were recorded for 15 min. For recording of distress calls, the tail was taken between thumb and index finger with gentle pressure and newborns lifted to elicit a minimum of ten audible distress calls (DCs) interspersed with ultrasounds (USDs). Recordings were performed with a calibrated condenser microphone (Bruel and Kjaer, Model 4135)

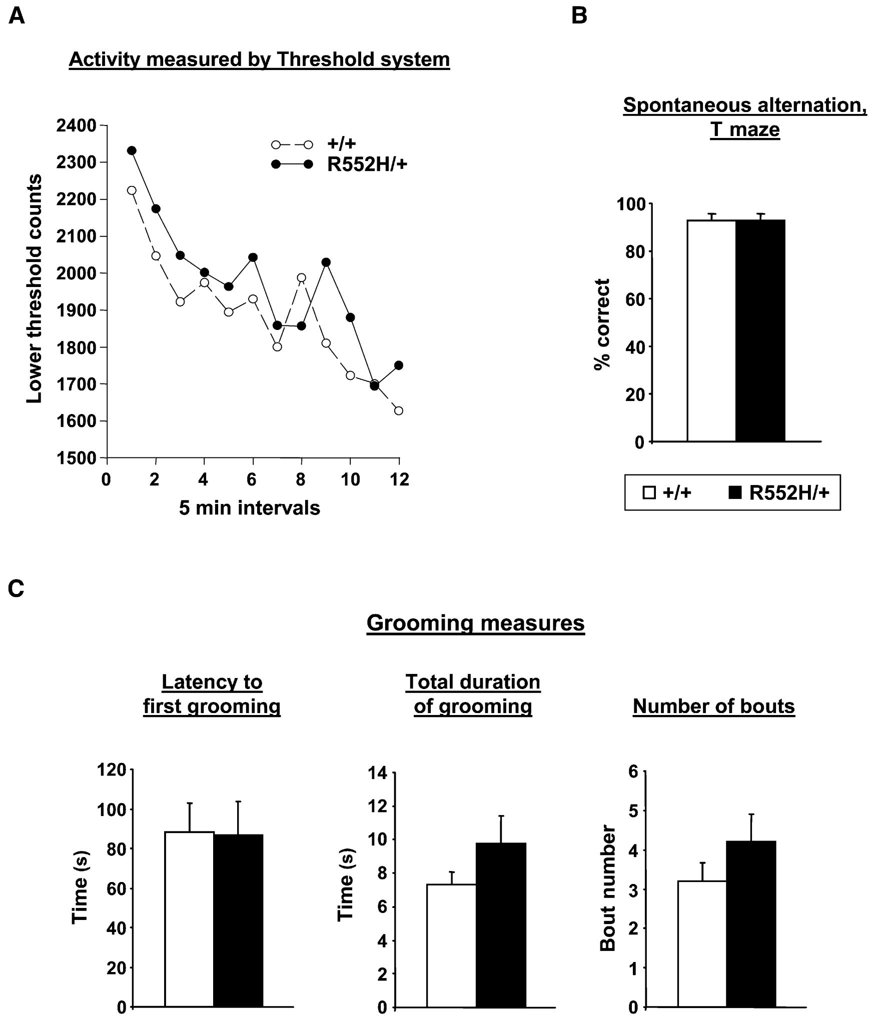

Figure S5. Behavior of *Foxp2-R552H* Heterozygous Mice

(A) Heterozygotes (n = 17) do not differ from wild-type littermates (n = 17) in spontaneous locomotor activity in a home-cage environment, assessed with a threshold activity system (Med Associates).

(B) R552H heterozygotes (n = 17) perform similarly to wild-type mice (n = 17) on tests of spontaneous alternation in the T maze (mean ± SEM).

(C) R552H heterozygotes (n = 10) do not differ from wild-type littermates (n = 10) on measures of grooming (mean ± SEM).

with preamplifier (Bruel and Kjaer, Model 2633) positioned about 8 cm above the mouse. The microphone output was high-pass filtered (Kemo VBF 10M, 132 dB/octave, 20 kHz high pass for USIs, 500 Hz high pass for DCs and USDs). The output of the filter was amplified (Bruel and Kjaer measuring amplifier, Model 2636, 40 dB amplification for USIs and 70 dB amplification for USDs and DCs) and recorded (Toshiba notebook CPU, 500 kHz DAQCard-6062E National Instruments, SIGNAL software version 4.1) with a gain of 10.0 and a sampling rate of 357143 Hz.

For measurement of peak sound pressure level (SPL) of the newborn calls, a calibration measurement was performed. Synthesized calls were recorded as described above for DCs, USDs, and USIs, and their SPL in decibels shown on the display of the measuring amplifier was synchronously noted. The SPLs were 80 dB for the distress calls, 85 dB for USDs, and 60 dB for USIs. The corresponding voltages of the synthesized calls were noted, calculated in rms values, and served for comparison with the voltages of the sampled natural calls of newborns.

All behavioral studies were performed with the experimenter blind for the genotype.

Table S1. Behavior of *Foxp2-R552H* Heterozygous Mice Compared to Wild-Type Littermates

	Wild-Type (+/+)		Heterozygous (R552H/+)		
	Mean ± SEM	n	Mean ± SEM	n	p
Open Field					
Total activity (cm)	2704 ± 166	8	2740 ± 144	12	0.89
Centre time (s)	13.2 ± 2.3	8	9 ± 1.4	12	0.17
Elevated Plus Maze					
Open arm dist (cm)	119 ± 23	9	106 ± 59	11	0.85
Open arm entry (#)	4 ± 0.6	9	3 ± 1.01	11	0.44
Open arm time (s)	24 ± 4.7	9	17 ± 9.2	11	0.50
Open arm latency (s)	76 ± 27.4	9	74.7 ± 27.6	11	0.95
Activity (Photocell)					
Total ambulation	1224 ± 80	9	1145 ± 113	12	0.60
Fine movement	263 ± 12.2	9	265 ± 12	12	0.89

Heterozygous R552H mice and wild-type littermates show similar baseline anxiety (mean ± SEM, t test) in open-field and elevated-plus-maze tests, both unconditioned tests of anxiety. In addition, there is no effect of genotype on spontaneous activity, assessed with a photoactivity system (San Diego Instruments).

Supplemental References

S1. Quwailid, M.M., Hugill, A., Dear, N., Vizor, L., Wells, S., Horner, E., Fuller, S., Weedon, J., McMath, H., Woodman, P., et al. (2004). A gene-driven ENU-based approach to generating an allelic series in any gene. Mamm. Genome *15*, 585–591.

S2. Augustin, M., Sedlmeier, R., Peters, T., Huffstadt, U., Kochmann, E., Simon, D., Schoniger, M., Garke-Mayerthaler, S., Laufs, J., Mayhaus, M., et al. (2005). Efficient and fast targeted production of murine models based on ENU mutagenesis. Mamm. Genome *16*, 405–413.

S3. Coghill, E.L., Hugill, A., Parkinson, N., Davison, C., Glenister, P., Clements, S., Hunter, J., Cox, R.D., and Brown, S.D. (2002). A gene-driven approach to the identification of ENU mutants in the mouse. Nat. Genet. *30*, 255–256.

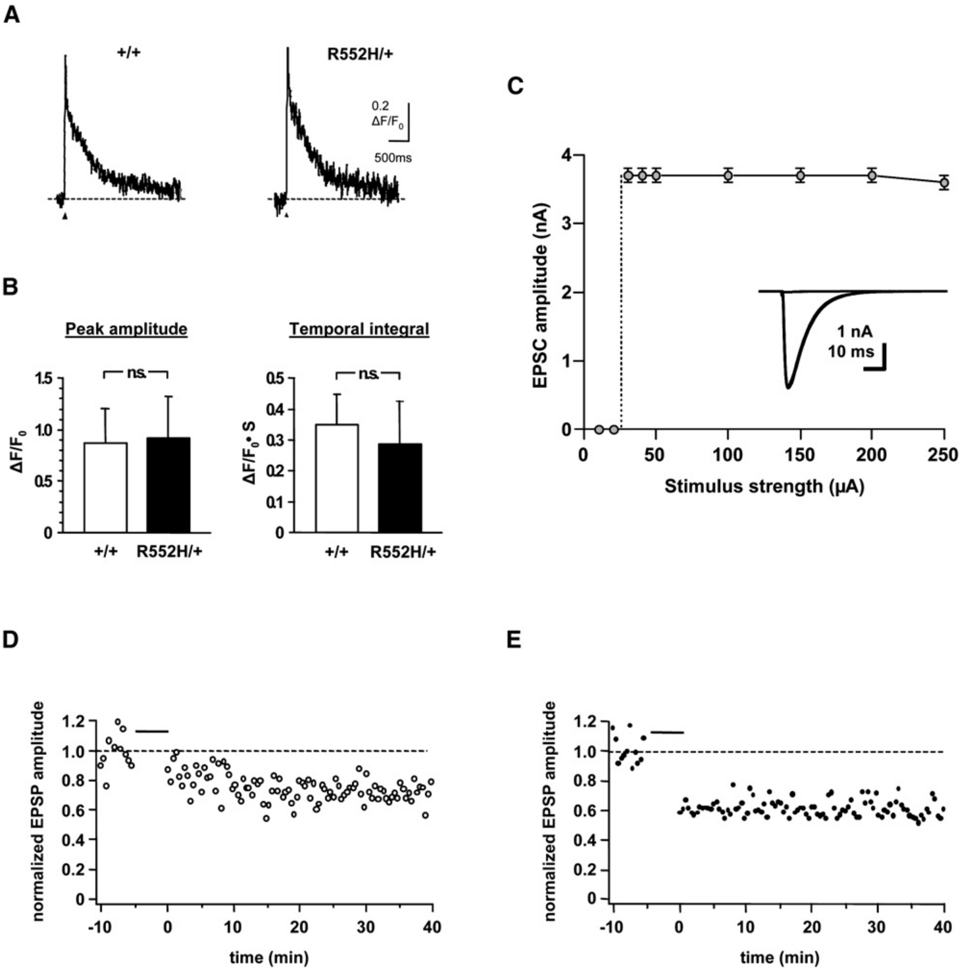

Figure S6. Cerebellar Electrophysiology in *Foxp2-R552H* Heterozygous Mice

(A) CF-evoked Ca^{2+} transients in distal dendrites (arrowhead: time point of stimulation). Representative traces from wild-type (left) and R552H heterozygous (right) mice.

(B) Summary data of the peak amplitude (left) and integral (right) of CF-evoked Ca^{2+} transients, indicating a grossly normal cerebellar synaptic circuitry in R552H heterozygotes (n.s. indicates not significant, mean ± SD, n = 5 mice/group).

(C) EPSC amplitudes (mean ± SD) evoked by stimulation of a CF in a PC from an R552H heterozygous mouse at increasing stimulus strength. The recording was performed in the presence of a submaximal concentration of the AMPA-receptor blocker CNQX. The dashed line represents the threshold at which EPSCs could be evoked. The inset shows the nine corresponding EPSCs superimposed (average of five responses at each stimulation strength). Stimulus artifacts were clipped for clarity. Note the all-or-none behavior of the responses, again consistent with undisturbed cerebellar synaptic circuitry.

(D) Example of LTD data in a PC from a wild-type mouse. Each data point represents the average of five synaptic responses. The black bar indicates the induction period (300 combined CF-PF stimulations at 1 Hz).

(E) Example of LTD data in a PC from an R552H heterozygous mouse. See Figure 3D in the main text for summary data and representative traces.

S4. Keays, D.A., Clark, T.G., Campbell, T.G., Broxholme, J., and Valdar, W. (2007). Estimating the number of coding mutations in genotypic and phenotypic driven N-ethyl-N-nitrosourea (ENU) screens: Revisited. Mamm. Genome *18*, 123–124.

S5. French, C.A., Groszer, M., Preece, C., Coupe, A.M., Rajewsky, K., and Fisher, S.E. (2007). Generation of mice with a conditional Foxp2 null allele. Genesis *45*, 440–446.

S6. Gerdeman, G.L., Ronesi, J., and Lovinger, D.M. (2002). Postsynaptic endocannabinoid release is critical to long-term depression in the striatum. Nat. Neurosci. *5*, 446–451.

S7. Edwards, F.A., Konnerth, A., Sakmann, B., and Takahashi, T. (1989). A thin slice preparation for patch clamp recordings from neurones of the mammalian central nervous system. Pflugers Arch. *414*, 600–612.

S8. Konnerth, A., Llano, I., and Armstrong, C.M. (1990). Synaptic currents in cerebellar Purkinje cells. Proc. Natl. Acad. Sci. USA *87*, 2662–2665.

S9. Karachot, L., Kado, R.T., and Ito, M. (1994). Stimulus parameters for induction of long-term depression in in vitro rat Purkinje cells. Neurosci. Res. *21*, 161–168.

S10. Crusio, W.E., and Schmitt, A. (1998). A multivariate quantitative-genetic analysis of behavioral development in mice. Dev. Psychobiol. *32*, 339–351.

S11. De Bono, J.P., Adlam, D., Paterson, D.J., and Channon, K.M. (2006). Novel quantitative phenotypes of exercise training in mouse models. Am. J. Physiol. Regul. Integr. Comp. Physiol. *290*, R926–R934.

S12. Guenther, K., Deacon, R.M., Perry, V.H., and Rawlins, J.N. (2001). Early behavioural changes in scrapie-affected mice and the influence of dapsone. Eur. J. Neurosci. *14*, 401–409.

S13. Deacon, R.M., and Rawlins, J.N. (2006). T-maze alternation in the rodent. Nat. Protocols *1*, 7–12.

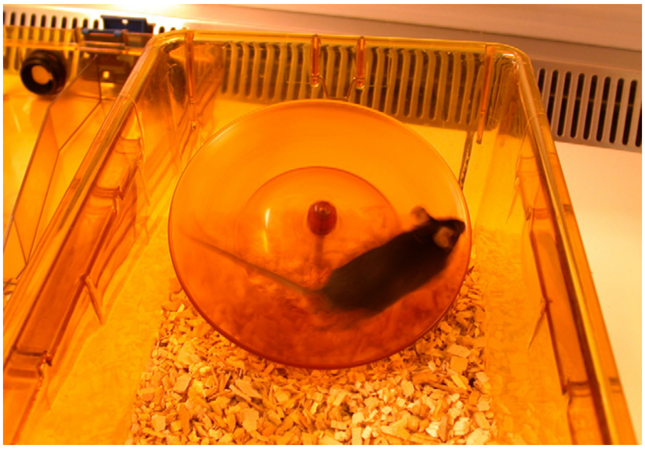

Figure S7. Voluntary Running-Wheel System

Photograph of tilted running-track system used for home-cage assessments of voluntary motor-skill learning.
